# 

**Published:** 1879-02

**Authors:** 


FEBRUARY, 1879.

### THE AMERICAN JOURNAL OF DENTAL SCIENCE,

EDITED BY

F. J. S. GORGAS, M. D., D. D. S.,

AND

JAMES B. HODGKIN, D. D. S.

S.G.O.L. INDEXED

PUBLISHED MONTHLY.

$2.50 PER ANNUM.

VOL. 12.—THIRD SERIES.—No. 10.

BALTIMORE:

SNOWDEN & COWMAN.

LONDON:
TRUBNER & CO., 60, PATERNOSTER ROW.

1879.

PAYABLE IN ADVANCE.

